# The Effect in Renal Function and Vascular Decongestion in Type 1 Cardiorenal Syndrome Treated with Two Strategies of Diuretics, a Pilot Randomized Trial

**DOI:** 10.1186/s12882-021-02637-y

**Published:** 2022-01-03

**Authors:** Jonathan S. Chávez-Iñiguez, Miguel Ibarra-Estrada, Sergio Sánchez-Villaseca, Gregorio Romero-González, Jorge J. Font-Yañez, Andrés De la Torre-Quiroga, Andrés Aranda-G de Quevedo, Alexia Romero-Muñóz, Pablo Maggiani-Aguilera, Gael Chávez-Alonso, Juan Gómez-Fregoso, Guillermo García-García

**Affiliations:** 1 Servicio de Nefrología, Hospital Civil de Guadalajara Fray Antonio Alcalde, Guadalajara, Jalisco Mexico; 2 Universidad de Guadalajara, Centro Universitario de Ciencias de la Salud CUCS, Hospital 278, CP 44240 Guadalajara, Jalisco Mexico; 3 Unidad de Terapia Intensiva, Hospital Civil de Guadalajara Fray Antonio Alcalde, Guadalajara, Jalisco Mexico; 4 Departamento de Nefrología, Clínica Universidad de Navarra, Pamplona, Spain

**Keywords:** Acute kidney injury, Cardio-renal syndrome, Congestive heart failure, Diuretics, Diuresis

## Abstract

**Aim:**

The main treatment strategy in type 1 cardiorenal syndrome (CRS1) is vascular decongestion. It is probable that sequential blockage of the renal tubule with combined diuretics (CD) will obtain similar benefits compared with stepped-dose furosemide (SF).

**Methods:**

In a pilot double-blind randomized controlled trial of CRS1 patients were allocated in a 1:1 fashion to SF or CD. The SF group received a continuous infusion of furosemide 100 mg during the first day, with daily incremental doses to 200 mg, 300 mg and 400 mg. The CD group received a combination of diuretics, including 4 consecutive days of oral chlorthalidone 50 mg, spironolactone 50 mg and infusion of furosemide 100 mg. The objectives were to assess renal function recovery and variables associated with vascular decongestion.

**Results:**

From July 2017 to February 2020, 80 patients were randomized, 40 to the SF and 40 to the CD group. Groups were similar at baseline and had several very high-risk features. Their mean age was 59 ± 14.5 years, there were 37 men (46.2%). The primary endpoint occurred in 20% of the SF group and 15.2% of the DC group (*p* = 0.49). All secondary and exploratory endpoints were similar between groups. Adverse events occurred frequently (85%) with no differences between groups (*p* = 0.53).

**Conclusion:**

In patients with CRS1 and a high risk of resistance to diuretics, the use of CD compared to SF offers the same results in renal recovery, diuresis, vascular decongestion and adverse events, and it can be considered an alternative treatment. ClinicalTrials.gov with number NCT04393493 on 19/05/2020 retrospectively registered.

**Supplementary Information:**

The online version contains supplementary material available at 10.1186/s12882-021-02637-y.

## Introduction

Heart performance and kidney function are strictly interconnected through a variety of pathways, including perfusion, filling pressure and neurohormonal activity [1]. Cardiorenal type 1 syndrome (CRS1) is characterized by a rapid worsening of cardiac function leading to acute kidney injury (AKI) [2]. In a meta-analysis, CRS1 occurred in one-fifth of patients with cardiac disease and increased the risk of death by 5 times [3]. Accumulating clinical evidence supports a key role for venous congestion in this syndrome [4]. Decongestion is the primary therapeutic goal in the majority of patients with acute decompensated heart failure (ADHF) [5]. The mainstay of therapy to obtain fluid removal is intravenous (IV) loop diuretics, mainly furosemide [6]. Unfortunately, an efficient diuretic response is not observed in many patients who present with ADHF or some of them develop resistance; therefore, synergizing the diuretic effect with thiazides [7] or spironolactone [8] has been explored, since blocking the renal tubule in different segments would potentiate the effect of furosemide. The sequential blockage of the renal tubule promotes greater natriuresis, urinary volume, effects observed even in patients at high risk of resistance to diuretics. So far, there are no clinical trials assessing sequential blockage of the renal tubule to promote decongestion by infused furosemide in CRS1 patients. Therefore, we conducted a double-blind clinical trial of patients with CRS1 in which we used a combined diuretic strategy compared to stepped furosemide, to promote renal recovery and the variables associated with vascular decongestion. Our hypothesis was that a combination of diuretics would be at least similar than stepped furosemide alone.

## Materials and Methods

### Study participants

This was a prospective pilot, phase II single-center double-blind randomized clinical trial that screened all consecutive patients admitted for acute decompensation (ADHF) and acute kidney injury (AKI), who met the criteria of cardiorenal type 1 syndrome (CRS1) and were evaluated by the Nephrology department at the Hospital Civil de Guadalajara Fray Antonio Alcalde, a large referral center that attends patients without health care insurance and low socioeconomic resources in Jalisco, México. Patients were enrolled from July 2017 to February 2020.

The research was conducted in accordance with the World Medical Association Declaration of Helsinki. The study was approved by the Institutional Review Board (HCG/CEI-0550/17), and all patients provided written informed consent. No funding was received to conduct this study. The trial was registered in ClinicalTrials.gov with number NCT04393493 on 19/05/2020.

### Definitions

Cardiorenal type 1 syndrome (CRS1) was defined according to the 2008 classification system by Ronco et al. [9]; to meet the criteria of CRS1, AKI was defined as an increase in serum creatinine (sCr) according to KDIGO [10], and acute decompensation heart failure (ADHF) was defined clinically [11]. Both criteria need to be present at the moment of the initial evaluation. The exclusion criteria were kidney transplantation, chronic kidney disease (CKD) grade 5, being receiving dialysis and pregnancy.

CKD was defined according to the KDIGO guideline [12]. The estimated glomerular filtration rate (eGFR) in ml/min/1.73 m^2^ was calculated according to the Chronic Kidney Disease Epidemiology Collaboration (CKD-EPI) [13]. Baseline eGFR was considered according to the last sCr (previous 3 months). All patients must have baseline sCr, considered in the last 6 months before hospitalization. Renal function recovery was defined as sCr return to baseline value at any point during the trial (complete recovery).

All comorbidities and clinical data were prospectively collected by direct contact with the patient during the first evaluation. Additional data were collected from medical records and the hospital electronic database.

The primary endpoint was to assess renal function recovery (*sCr return to baseline value*) after 96 h, and the secondary endpoints were a combination of variables related to vascular decongestion during the treatment and follow up, namely, the change in urinary output, sCr, sCr worsening, in-hospital mortality, mortality during follow up, dyspnea improvement, dyspnea improvement before day 3, time until dyspnea improvement, renal replacement therapy, change in electrolytes and acid-base status. As exploratory endpoints, we evaluated changes in copeptine levels, BNP, BNP reduction and intervention stop because of clinical improvement. Prespecified adverse events were reported. The sample size was for convenience, there are no clinical trials similar to this.

## Randomization and treatments assignments

Randomization was carried out by the investigators by a computer-based stratified randomization was generated (1:1), with the strata defined by sex. Allocation was done by the nephrology staff on a concealed opaque envelope until the beginning of the study. A double-blind, double-dummy design was used. All patients received a bolus of furosemide 80 mg every day, a low sodium diet (< 2.4 g sodium/day), and strict fluid control was prescribed (< 1000 ml/day).

The Stepped Furosemide (SF) group received a continuous daily infusion during 24 h. of furosemide 100 mg diluted in 100 ml of Hartmann solution during the first day, with daily incremental doses to 200 mg, 300 mg and 400 mg during the second, third and fourth day, respectively. This arbitrary strategy was considered “conventional” because reflect the common increases in furosemide that has been previously chosen, this strategy has been previously observed that only with an increase in the dose of furosemide the objective of vascular decongestion can be achieved and this study was designed before the recommendations to measure the efficiency of loop diuretics according to sodium and urinary volume, additionally 2 placebo capsules, with the intention of keeping the blind. The Combined Diuretics (CD) group was given a combination of diuretics trying to block different tubular segments, similar (but not equal) to the CARRESS-HF trial [14], including 4 consecutive days of oral chlorthalidone 50 mg, spironolactone 50 mg and continuous infusion of furosemide 100 mg diluted in 100 ml of Hartmann for 24 h, as shown in Supplemental Fig. 1. The choice of these 3 diuretics is justified under the hypothesis that sequential blockage of the renal tubule promotes effective vascular decongestion. In both groups, the assigned treatment strategy was continued until the signs and symptoms of congestion had been resolved or until the end of the trial (96 h). Inotropes, vasopressors, continuous positive airway pressure ventilation or management of any other comorbid condition were modified at the discretion of the attending physicians, who maintained constant communication with nephrology staff.

## Statistical analysis

Based on the Shapiro-Wilk test, continuous variables are reported as the means (standard deviation [SD]) if they were normally distributed, or medians (interquartile range [IQR]) if they were not normally distributed. These variables were compared between groups with Mann-Whitney or t-tests as appropriate. Categorical variables are expressed as proportions and were compared by x^2^ tests or Fisher’s exact test. The relative risk was calculated for all secondary outcomes, taking CD group as reference. For all tests, *p* values were two-sided, and a value < 0.05 was considered statistically significant. MedCalc Statistical Software (Ostend, Belgium. Ver 19.1.3) was used for statistical analysis and GraphPad Prism (California, USA. Ver 9.2.0) for graphics.

## Results

**Fig. 1:**
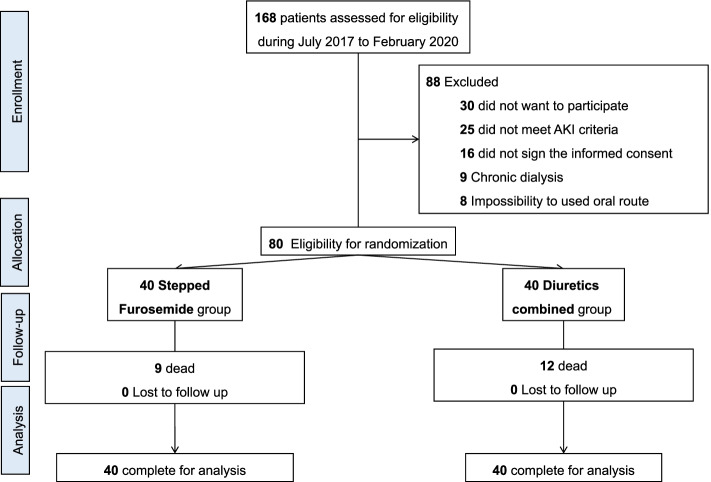
CONSORT diagram of allocation groups

During the study period from July 2017 to February 2020, 168 patients were assessed for eligibility, and 88 were excluded for not meeting the inclusion criteria. Therefore, 80 patients were randomized, 40 to the SF and 40 to the CD group, as shown in the diagram in Fig. 1, according to CONSORT 2010 [15]. No patients were lost to follow-up.

The demographic and clinical characteristics of the study participants are described in Table 1. Both groups were similar, with no significant differences between them. Median follow up was 182 (IQR 65) days. Mean age was 59 ± 14.5 years, there were 37 men (46.2%); and the patient population had several very high-risk features, including diabetes 55 (71.4) and hypertension 64 (80%); baseline sCr 2.9 mg/dL (2.3); CKD was present in 34 (44.2%), with an eGFR 28 ml/min/1.73 m^2^ (31); acute myocardial infarction in 16 (21.3%); chronic heart failure 50 (65.8%); systolic 130 (23) mmHg and diastolic 75 (13) mmHg blood pressure; proteinuria on dipstick 38 (64.4%); and BNP 2631 (1713) ng/dL. Medical management during their hospitalization was similar between the groups. Antibiotics were prescribed in 49 (64.3%), only 4 (5%) received 0.9% saline, and 56 (70.9%) Hartmann solution. Most patients were admitted to Internal Medicine and Cardiology departments 75 (93.7%), Table 1.

**Table 1:** Demographic and clinical characteristics of 80 CRS1 patients according to allocation group

	All patients *n* = 80	Stepped Furosemide *n* = 40	Combined Diuretics n = 40	p
Male (%)	37 (46.2)	19 (47.5)	18 (45)	0.82
Age (years)	59 ± 14.5	58 ± 14.5	59 ± 14.6	0.73
Comorbidities				
Diabetes (%)	55 (71.4)	25 (64.1)	30 (78.9)	0.15
Hypertension (%)	64 (80)	29 (74.4)	35 (92.1)	0.06*
Baseline sCr (mg/dL) (IQR)	2.9 (2.3)	3.1 (2.5)	2.8 (1.9)	0.58
CKD (%)	34 (44.2)	16 (41)	18 (47.4)	0.57
Baseline GFR (ml/min/1.73m^2^) (IQR)	28 (21)	31 (22)	28 (46)	0.98
Acute myocardial infraction (%)	16 (21.3)	8 (21.1)	8 (21.6)	0.95
Chronic heart failure (%)	50 (65.8)	26 (66.7)	24 (64.9)	0.86
Hypothyroidism (%)	8 (10.7)	6 (16.2)	2 (5.3)	0.15*
Arrythmia (%)	11 (14.3)	5 (12.8)	6 (15.8)	0.75*
Current smoker (%)	36 (46.8)	16 (41)	20 (52.6)	0.31
Vital signs and baseline laboratory results				
Heart rate (bpm) (IQR)	85 (26)	87 (19)	82 (24)	0.32
Oxygen saturation (%) (IQR)	94 (4)	95 (4)	94 (4)	0.63
Systolic blood pressure (mmHg) (SD)	130 ± 23	126 ± 23	134 ± 22	0.11
Diastolic blood pressure (mmHg) (SD)	75 ± 13	74 ± 12	77 ± 14	0.23
Uric acid (mg/dL) (IQR)	8.6 (2.2)	8.5 (1.4)	9.1 (3.3)	0.34
Proteinuria, dipstick (%)	38 (64.4)	20 (66.7)	18 (62.1)	0.71
Hematuria (%)	34 (57.6)	19 (63.3)	15 (51.7)	0.37
BNP (ng/dL) (SD)	2631 ± 1713	2501 ± 1669	2718 ± 1836	0.81
Copeptin (ng/dL) (IQR)	75 (121)	75 (178)	72 (66)	0.60
Management				
Antibiotics (%)	49 (65.3)	23 (60.5)	26 (70.3)	0.37
Blood Transfusion (%)	4 (5.4)	3 (8.1)	1 (2.7)	0.61*
Vasopressor (%)	5 (6.8)	3 (8.1)	2 (5.4)	0.67*
Inotropic (%)	2 (2.7)	1 (2.7)	1 (2.7)	1.0*
Diuretics (%)	16 (20)	10 (25)	6 (15)	0.26
Urinary volume (ml/ day) (IQR)	1266.57 (675)	1278.93 (675)	1235.38 (638)	0.56*
Saline 0,9% IV fluid (%)	4 (5)	2 (5)	2 (5)	1.0*
Hartmann IV fluid (%)	56 (70.9)	27 (67.5)	29 (74.4)	0.50
Department of admission				
Internal medicine (%)	47 (60.3)	24 (61.5)	23 (59)	0.81
Cardiology (%)	28 (35.9)	14 (35.9)	14 (35.9)	1.0
Intensive care unit (%)	1 (1.3)	0	1 (2.6)	0.49*
Surgical specialty (%)	2 (2.6)	1 (2.6)	1 (2.6)	1.0

IV, intravenous; CKD, chronic kidney disease; sCr, serum creatinine; GFR, glomerular filtration rate; SD, standard deviation; IQR, interquartile range. *Fisher’s exact test was used

### Clinical outcomes

**Fig. 2:**
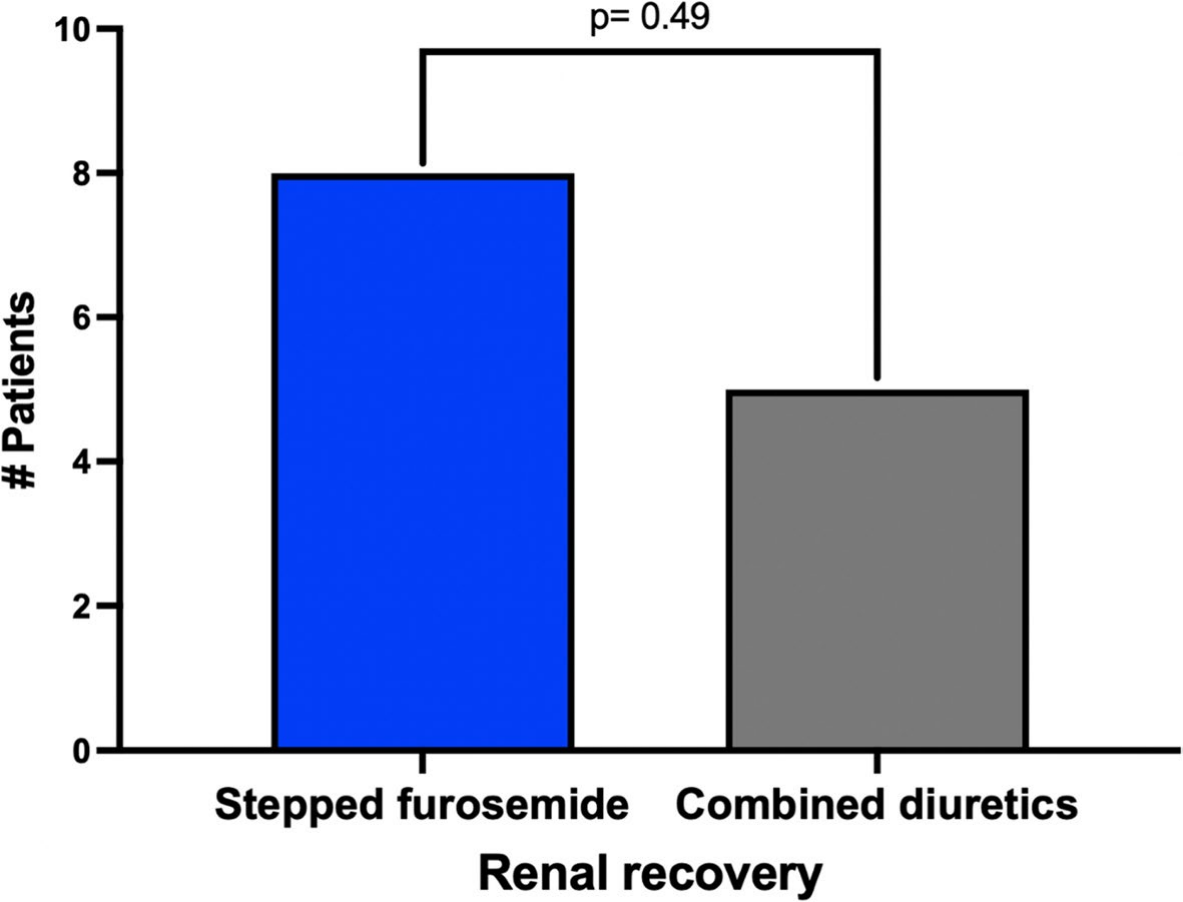
Primary endpoint Renal function recovery (*sCr return to baseline value*) in 80 patients with CRS1 according to allocation groups

The primary endpoint of renal function recovery (*sCr return to baseline value*) after 96 h occurred in 8 patients (20%) in the SF group and in 5 (15.2%) in the CD group (RR 1.5, 95% CI 0.4–5.2; *p* = 0.49, supplemental Table 1 and Fig. 2), During the 4 days of the study, the sCr increased but did not reach statistical significance when compared across subsequent days or between the study groups (supplemental Table 1).

**Fig. 3:**
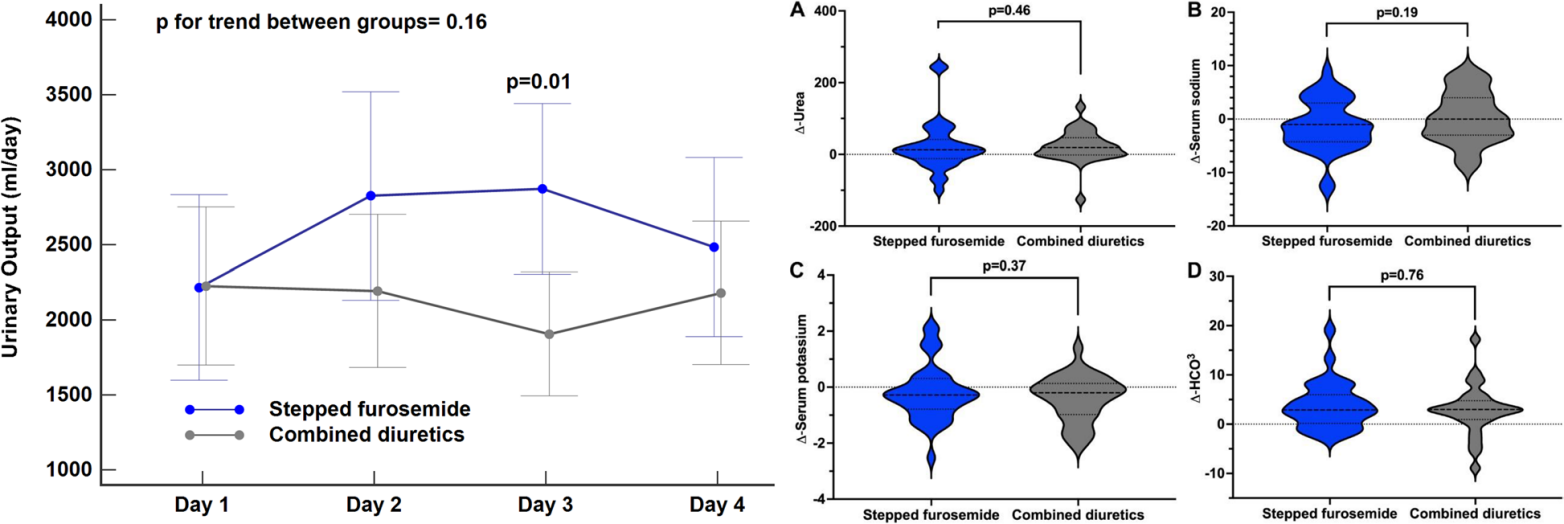
Secondary endpoint in 80 patients with CRS1 according to allocation groups. 3.1) Change in urinary output (ml). 3.2) Change in A, urea; B, serum sodium; C, serum potassium and D, serum bicarbonate

**Fig. 4:**
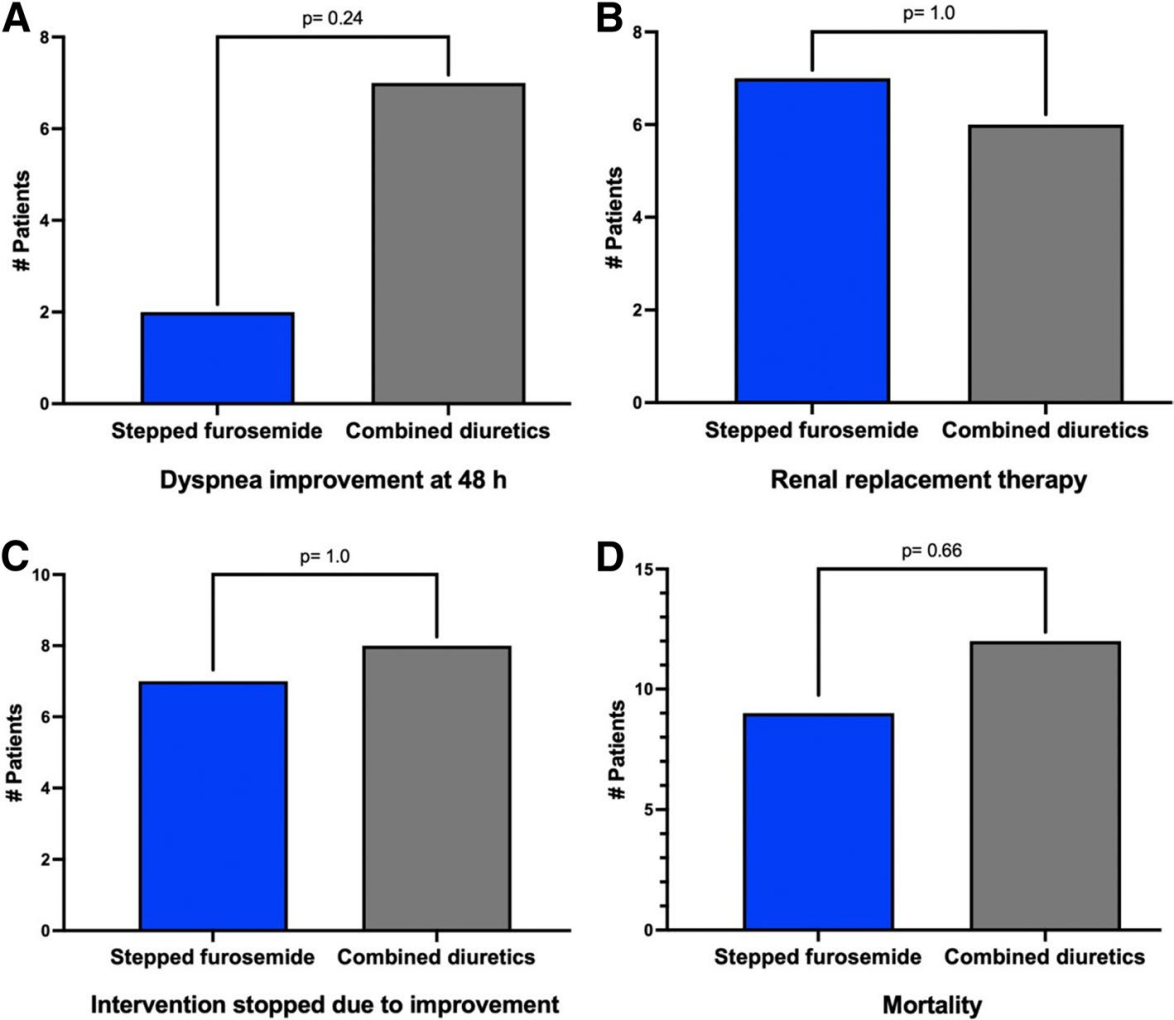
Clinical evolution in 80 patients with CRS1 according to allocation groups. A) Dyspnea improvement, B) Renal replacement therapy, C) Intervention stopped because improvement and D) mortality

All secondary endpoints were similar between groups as shown in supplemental Table 2 and Fig. 3. Increase in daily urinary output at 96 h with respect to the baseline were similar between groups, 125 ml (1662) in the SF group and 200 ml (988) in the CD group (*p* = 0.30); sCr increased by 0.02 mg/dL (0.9) in the SF group and by 0.2 mg/dL (0.52) in the CD group (*p* = 0.26). During the 4 days of the study, the daily urinary output increased but without reaching significance when compared between the days or between the study groups (supplemental Table 1). There were no significant differences in other prespecified secondary endpoints such as sCr worsening (*p* = 0.20), in-hospital mortality (p = 0.20), mortality at follow-up (*p* = 0.43), dyspnea improvement (*p* = 1.0), total days to dyspnea improvement (*p* = 0.51), or renal replacement therapy (p = 1.0), as shown in Fig. 4.

**Fig. 5:**
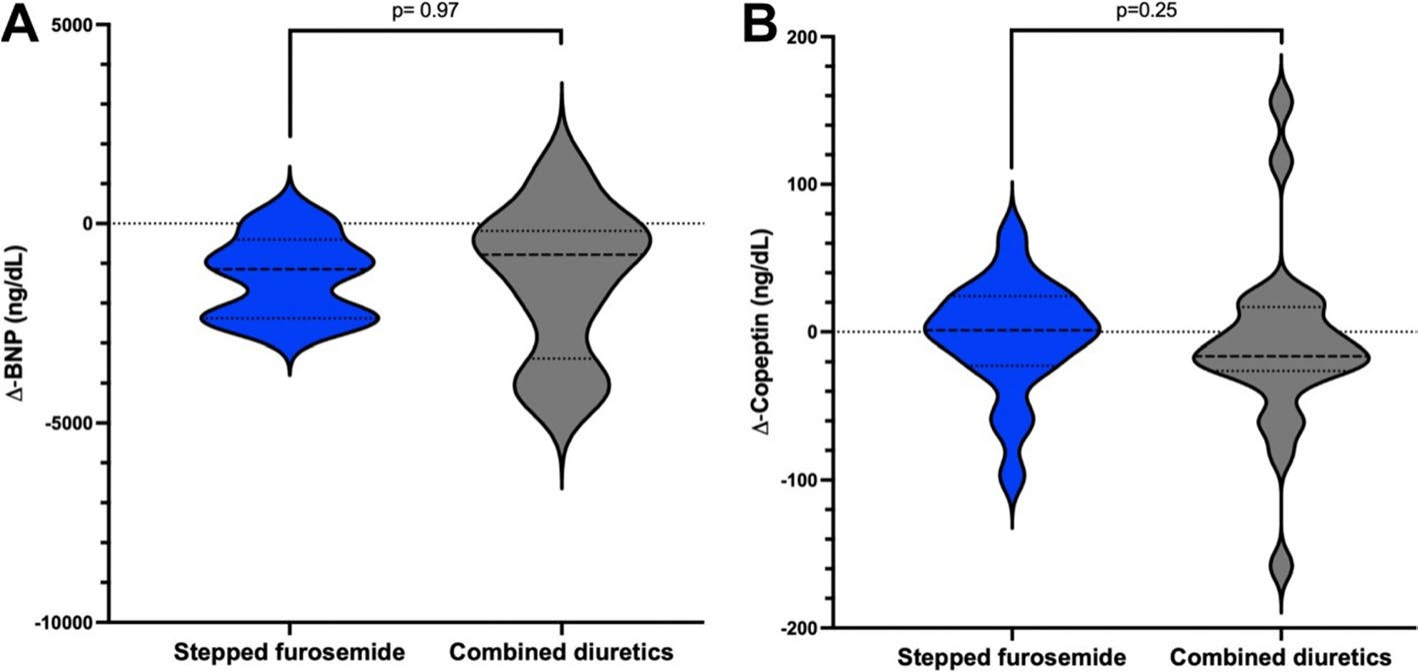
Exploratory analysis, changes in BNP (A) and copeptine levels (B) during the trial according to the allocation groups

We also found no significant differences in the exploratory endpoints between groups (supplemental Table 1 and Fig. 5). Copeptine decreased in a similar manner (*p* = 0.25) as BNP (*p* = 0.97). BNP reduction > 30% from the randomization value was achieved in 75% of all patients, 71.0% in the SF and 77.7% in the CD, with no difference between groups; intervention stopped because clinical improvement occurred in only 15% (p = 1.0).

Changes in urea, electrolytes and acid-base values during the trial are shown in supplemental Table 2 and Fig. 3. Briefly, both treatments increased serum urea, magnesium, pH, bicarbonate, pCO_2_ and lactate, but none of these were significantly different between groups.

Adverse events are shown in Table 2; any adverse event was present in 68 (85%) of patients, with no difference between groups (*p* = 0.53); metabolic alkalosis in 24 (30%), hypokalemia in 15 (18.8%), hyponatremia in 36 (45%), hypotension in 5 (6.2%), and no cases of hypomagnesemia were observed.

**Table 2:** Adverse events of Cardiorenal type 1 patients according to allocation groups during the study period

	All patients, n = 80	Stepped Furosemide, n = 40	Combined Diuretics n = 40	p value
Any adverse event (%)	68 (85)	35 (7.5)	33 (82.5)	0.53
Metabolic alkalosis (%)	24 (30)	12 (30)	12 (30)	0.60
Hypokalemia (%)	15 (18.8)	8 (20)	7 (17.5)	0.78
Hyponatremia (%)	36 (45)	18 (45)	18 (45)	1.0
Hypotension (%)	5 (6.2)	4 (10)	1 (2.5)	0.35

## Discussion

In this pilot randomized, double-blind clinical trial in 80 patients with high-risk CRS1, we found that CD compared with SF for 96 h offers the same effect in renal function recovery, clinical evolution and adverse effects. To the best of our knowledge, this is the first clinical trial that compared these 2 vascular decongestion strategies in this context.

Renal functional recovery occurred in only 13 (18.6%) of the entire sample (*p* = 0.49), with no significant difference between groups. Before interpreting the diuretic strategies as ineffective due to the low frequency of renal recovery of CRS1, the following points should be considered: first, we included patients with low kidney function (eGFR 28 ml/min/1.73 m^2^), much lower than other clinical trials performed in this context [16,17], and they had a higher frequency of diabetes (71.4%), hypertension (80%) and proteinuria (64%). All of these characteristics are considered as high risk of resistance to diuretics and are strong predictors of a poor clinical evolution [18]. Second, it has been shown in patients with CRS1 that the absence of renal recovery, or even its worsening during the treatment of vascular decongestion with diuretics, is associated with a good clinical evolution [19,20,21]. In our study, the sCr tended to increase (0.08 mg/dL), and 61.1% of cases had worsened kidney function, and an increase in urea was also observed (14 mg/dL). Acute decreases in renal function after ADHF undergoing aggressive decongestion with high-dose loop diuretics does not necessarily reflect structural injury to the kidneys [22]. Third, this “lack of effectiveness” to renal recovery was accompanied by a reduction in BNP (− 1365 ± 1574), and even during treatment it was possible to decrease BNP > 30% in 36% of cases, an event that can be interpreted as effective decongestion, that is significantly associated with greater survival in the median long term [23], and guiding the treatment of these patients with natriuretic peptides has been proven to decrease their probability of hospitalizations by 20% and their probability of dying by 13% [24].

Urinary output was equivalent in both diuretic strategies. This outcome may not represent a negative result of our study, since it could be interpreted as a strategy to save furosemide by avoiding doubling its dose every 24 h. Potential mechanisms for worse outcomes with high doses of loop diuretics have been described, including stimulation of the renin-angiotensin-aldosterone system (RAAS) and sympathetic nervous system, electrolyte disturbances, and deterioration of renal function [25]. There is a physiological plausibility to think that in CRS1 patients who already had an activated neurohormonal state, the strategy of blocking the renal tubule with different diuretics at not so high doses sounds reasonable. Also, because the main cause of resistance to loop diuretics is an adaptation of the distal and collector tubules to maximize the absorption of sodium and chloride [26,27,28], the use of thiazides could improve the response to treatment. They inhibit the sodium-chloride cotransporter (NCC) in the distal convoluted tubule. Regarding spironolactone, its diuretic action in the collecting tubule occurs by inhibiting the synthesis and apical expression of the ENaC channel while inhibiting the excretion of potassium through ROMK channels [29].

Recently, a pilot study on the use of spironolactone in subjects with diuretic-resistant ADHF reported clinically significant weight loss and reduced dyspnea without associated worsening hyperkalemia or renal function. In the ATHENA-HF trial, spironolactone (25 mg/day) showed no difference in symptoms or urine output compared to placebo; however, the follow-up was only 96 h (this drug only begins its effect between 48 and 72 h) [8], and this “delayed” effect of spironolactone could explain why our CD group had the same urine output as the SF group. It is possible that with more days of follow-up, the synergistic effect of spironolactone could be reflected.

Decreasing the feeling of dyspnea is one of the most important goals in treating CRS1, and Frea et al. [16] reached this goal in only 48% of the cases after 3 days of management. Our results differ since we found that the median number of days to find relief of dyspnea was 4 days, and moreover, our infusion dose of furosemide reached 400 mg on day 4 and the maximum dose in the infusion arm of the DRAIN study was 216 mg [16].

In our study, the same variations in electrolytes and acid-base status were observed in both groups, which can be considered clinically nonsignificant. We observed a similar decrease in potassium (− 0.29 ± − 0.9 mEq/L, *p* = 0.30), an expected alteration due to the doses of diuretics that were used in the SF arm, but it is striking that in the CD arm, this event occurred despite consuming spironolactone 50 mg per day and having a low kidney function (GFR 28 ml/min/1.73 m^2^). Other important electrolytes such as magnesium, chloride and sodium did not change significantly. As expected, bicarbonate and pH increased in both groups but only by 2.9 and 0.03, respectively, changes that have no relevant clinical impact.

Adding spironolactone to CRS1 has previously been shown to be safe. There was no incidence of hyperkalemia even though 24% of the patients in the high-dose spironolactone arm had CKD (eGFR 45–75 ml/min/1.73 m^2^) (8), so it could be started early in decongestive therapy, especially in the case of hypokalemia secondary to the use of loop diuretics or thiazides. There is less available evidence about the actual initiation of spironolactone in the setting of ADHF.

We found that adverse events are frequent during these diuretic strategies applied for vascular decongestion, since they were present in 85% of the patients. None were significantly different between groups. In order of frequency, the adverse events were: metabolic alkalosis, hyponatremia, hypokalemia and hypotension. In an analysis of 3 clinical trials of vascular decongestion in 744 patients with CRS1, it was described that bicarbonate at hospital discharge was increased in the patients in all 3 trials by 29 mEq/L (27–29), which was associated with an increased risk of hospitalization (30). They also report that every 1 mEq of increase in serum sodium was associated with a 5% less risk of hospitalization (30). Therefore, it is suggested that at least every 24 h these laboratory variables should be monitored and that any electrolyte and acid-base disorders are corrected as necessary.

### Limitations and strengths

Our results must be interpreted with caution, as this was a pilot single-center study without an a priori calculation of sample size due to the lack of literature to estimate an expected minimal clinically important difference between groups, so a type II error cannot be ruled-out; for instance, according to the observed difference in the primary outcome between groups, the *post-hoc* calculated power was 50% in our sample, maintaining an α-error probability of 5%. There was also a lack of hemodynamic, body weight changes and ultrasonographic measurements for a better estimate of intravascular volume and a lack of biomarkers of renal tubular damage that reflect true kidney injury. There was also a lack of reporting of other variables that could be relevant to our objectives.

The strengths of this study lie in its design, the adequate adherence of the allocation groups, and the length of follow-up. To our knowledge, this is the only clinical trial that has compared these two diuretic strategies in patients with CRS1.

## Conclusion

In patients with CRS1 and a high risk of resistance to diuretics, the strategy of CD (furosemide, chlorthalidone and spironolactone) compared to SF (furosemide in incremental infusion) offers the same frequency of renal recovery, diuresis, vascular decongestion and adverse events, so it can be considered as an alternative, especially in cases where it is not considered advisable to increase the dose of furosemide. Additional studies comparing these 2 strategies should be carried out with a larger number of patients, more days of treatment, and follow-up of immediate effects.

## Supplementary Information

**Supplemental figure 1.** Assignation and intervention of the study trial. (PPTX 71 kb)

**Supplemental Table 2.** CRS1 according to allocation groups during the study period.

**Supplemental Table 3.** Urea, electrolytes and acid-base evolution of CRS1 patients according to allocation groups during the study period.

## Abbreviations

ADHF: acute decompensated heart failure
AKI: acute kidney injury.
BNP: brain natriuretic peptide.
CD: combined diuretics.
CKD-EPI: Chronic Kidney Disease Epidemiology Collaboration.
CKD: chronic kidney disease.
CRS1: cardiorenal syndrome 1.
eGFR: estimated glomerular filtration rate.
ENaC: epithelial sodium channel.
IQR: Interquartile range.
KDIGO: kidney disease improves global outcomes.
mEq: milli equivalents.
NCC: sodium-chloride cotransporter.
pCO_2_: carbon dioxide blood pressure.
RAAS: renin-angiotensin-aldosterone system.
sCr: serum creatinine.
SD: standard deviation.
SF: Stepped Furosemide.
ROMK: renal outer medullary K.
RR: risk ratio.
