# Supplementary material for: The Effect in Renal Function and Vascular Decongestion in Type 1 Cardiorenal Syndrome Treated with Two Strategies of Diuretics, a Pilot Randomized Trial
Source: BMC Nephrol. 2022 Jan 3;23:3. doi: 10.1186/s12882-021-02637-y (PMC8722345; doi:10.1186/s12882-021-02637-y)
Supplement: Supplementary file 1 — Supplemental figure 1. Assignation and intervention of the study trial. (PPTX 71 kb) [file 12882_2021_2637_MOESM1_ESM.pptx]

## Slide 1
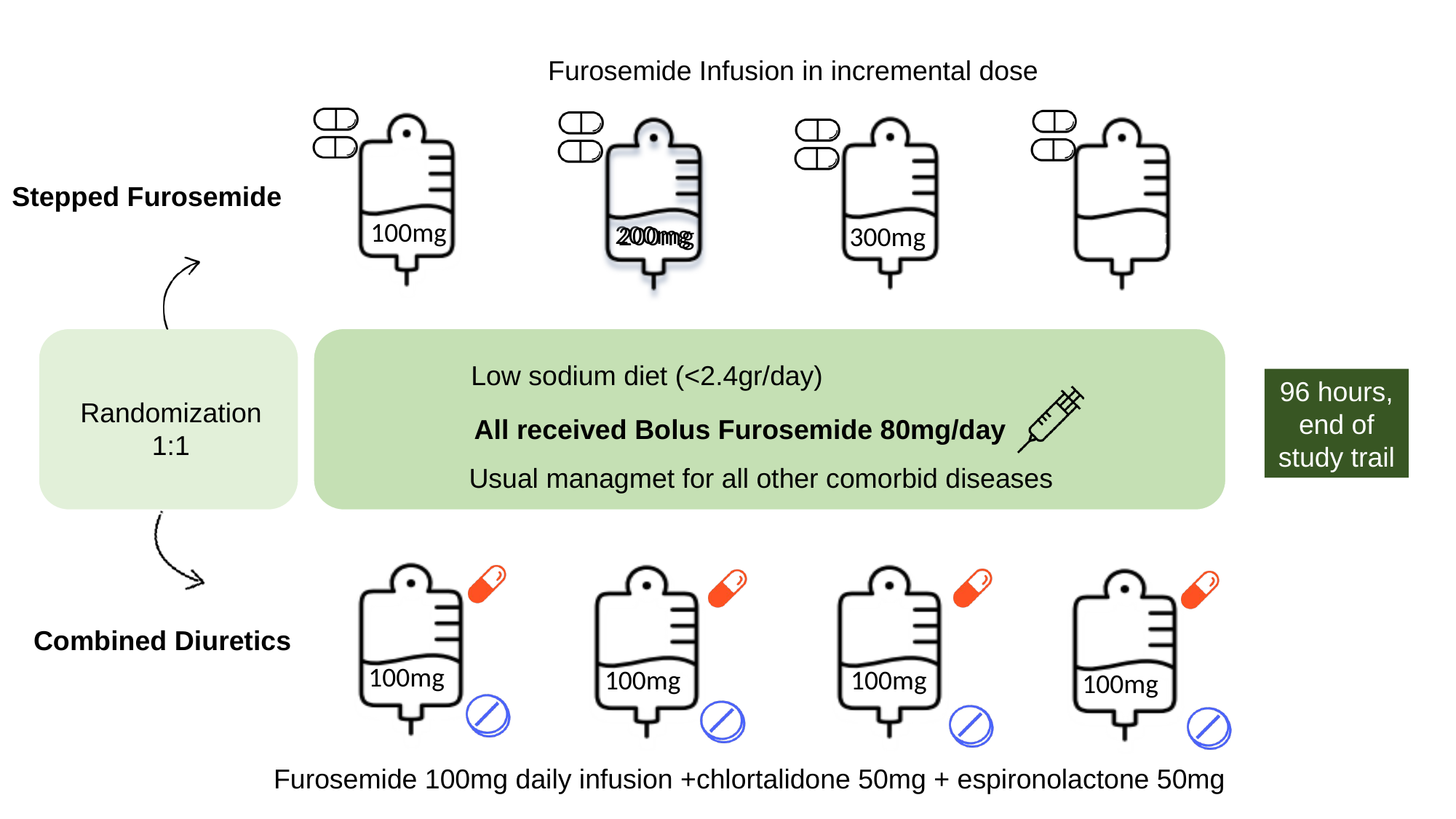

Furosemide Infusion in incremental dose
Stepped Furosemide
100mg
200mg
200mg
400mg
300mg
Low sodium diet (<2.4gr/day)
96 hours, end of study trail
Randomization
1:1
All received Bolus Furosemide 80mg/day
Usual managmet for all other comorbid diseases
Combined Diuretics
100mg
100mg
100mg
100mg
Furosemide 100mg daily infusion +chlortalidone 50mg + espironolactone 50mg
