# Supplementary material for: The Effect in Renal Function and Vascular Decongestion in Type 1 Cardiorenal Syndrome Treated with Two Strategies of Diuretics, a Pilot Randomized Trial
Source: BMC Nephrol. 2022 Jan 3;23:3. doi: 10.1186/s12882-021-02637-y (PMC8722345; doi:10.1186/s12882-021-02637-y)
Supplement: Supplementary file 2 — Supplemental Table 2. CRS1 according to allocation groups during the study period. [file 12882_2021_2637_MOESM2_ESM.docx]

**Supplemental Table 1. CRS1 patients according to allocation groups during the study period.**

|  | All patients,  n = 80 | Stepped Furosemide  n=40 | Combined Diuretics  n=40 | p value | RR (95% CI) |
| --- | --- | --- | --- | --- | --- |
| **Primary endpoint** |  |  |  |  |  |
| Renal function recovery (*sCr return to baseline value*) (%) | 13 (16.8) | 8 (20) | 5 (15.2) | 0.49 | 1.5 (0.4-5.2) |
| **Secondary endpoints** |  |  |  |  |  |
| Δ Urinary output (ml)  (IQR) | 185 (1,100) | 125 (1,662) | 200 (988) | 0.30 | NA |
| Δ sCr at 96 h (mg/dL) (IQR) | 0.08 (0.7) | 0.02 (0.9) | 0.2 (0.52) | 0.26 | NA |
| sCr worsening at 96 h (%) | 44 (61.1) | 20 (54.1) | 24 (68.6) | 0.20 | 0.53 (0.20-1.41) |
| In hospital mortality (%) | 8 (10) | 4 (10) | 4 (10) | 1.0 | 1.0 |
| Mortality during follow up (%) | 21 (29.2) | 9 (25) | 12 (33.3) | 0.43 | 0.66 (0.23-1.85) |
| Dyspnea improve (%) | 54 (67.5) | 27 (67.5) | 27 (67.5) | 1.0 | 1.0 |
| Dyspnea improvement before day 3 (%) | 9 (11.3) | 2 (5) | 7 (17.5) | 0.15 | 0.24 (0.04-1.27) |
| Time in days until dyspnea improved (IQR) | 4 (1) | 4 (1) | 4 (1.8) | 0.51 | NA |
| Renal replacement therapy (%) | 13 (17.1) | 7 (18.4) | 6 (15.8) | 1.0 | 1.2 (0.3-3.9) |
| **Exploratory endpoints** |  |  |  |  |  |
| Δ Copeptin at 96 h (ng/dL) (IQR) | -4.5 (41) | 1.1 (41) | -16 (39) | 0.25 | NA |
| Δ BNP at 96 h (ng/dL) (SD) | -1365 ± 1574 | -1344 ± 1041 | -1378 ± 1904 | 0.97 | NA |
| BNP reduction >30% at 96 h (%) | 12 (75) | 5 (71.4) | 7(77.7) | 0.49 | NA |
| Intervention stopped because improve (%) | 15 (18.8) | 7 (17.5) | 8 (20) | 1.0 | 0.84 (0.27-2.61) |

Δ, change at 96 h; BNP, brain natriuretic peptide; sCr serum creatinine; IQR, interquartile range; SD, standard deviation.
