# Supplementary material for: The Effect in Renal Function and Vascular Decongestion in Type 1 Cardiorenal Syndrome Treated with Two Strategies of Diuretics, a Pilot Randomized Trial
Source: BMC Nephrol. 2022 Jan 3;23:3. doi: 10.1186/s12882-021-02637-y (PMC8722345; doi:10.1186/s12882-021-02637-y)
Supplement: Supplementary file 3 — Supplemental Table 3. Urea, electrolytes and acid-base evolution of CRS1 patients according to allocation groups during the study period. [file 12882_2021_2637_MOESM3_ESM.docx]

**Supplemental Table 2. Urea, electrolytes and acid-base evolution of CRS1 patients according to allocation groups during the study period.**

|  | All patients,  n = 80 | Stepped Furosemide,  n=40 | Combined Diuretics,  n=40 | p |
| --- | --- | --- | --- | --- |
| Δ sUrea (mg/dL) (IQR) | 14 (49) | 13 (48) | 18 (46) | 0.46 |
| Δ sSodium (mEq/L) (SD) | -0.5 ± 4.7 | -1.2 ± 4.7 | 0.2 ± 4.7 | 0.19 |
| Δ sPotassium (mEq/L) (SD) | -0.29 ± -0.9 | -0.2 ± 0.9 | -0.4 ± 0.8 | 0.37 |
| Δ sChloride (mEq/L) (SD) | -0.2 ± 0.9 | -0.6 ± 6.4 | -0.4 ± 4.7 | 0.05 |
| Δ sMagnessium (mEq/L) (SD) | 0.03 ± 0.2 | 0.07 ± 0.16 | -0.04 ± 0.2 | 0.23 |
| Δ sCalcium (mEq/L) (IQR) | 0 | 0.15 (-0.4-0.6) | -0.05 (-0.2-0.3) | 0.56 |
| Δ pH value (SD) | 0.03 ± 0.06 | 0.03 ± 0.07 | 0.02 ± 0.04 | 0.77 |
| Δ Bicarbonate (IQR) | 2.9 (5.8) | 2.9 (5.8) | 3 (3.6) | 0.76 |
| Δ pCO_2_ (IQR) | 1 (8) | 1 (8.7) | 3 (7.5) | 0.62 |
| Δ Lactate (IQR) | 0.05 (0.7) | 0 (0.7) | 0.1 (0.5) | 0.88 |

S, serum; Δ, change at 96 h; pCO^2^, partial pressure of oxygen; IQR, interquartile range; SD, standard deviation.
